# Unmanipulated haploidentical hematopoietic stem cell transplantation for pediatric *de novo* acute megakaryoblastic leukemia without Down syndrome in China: A single-center study

**DOI:** 10.3389/fonc.2023.1116205

**Published:** 2023-02-16

**Authors:** Junbin Huang, Guanhua Hu, Pan Suo, Lu Bai, Yifei Cheng, Yu Wang, XiaoHui Zhang, KaiYan Liu, YuQian Sun, LanPing Xu, Jun Kong, ChenHua Yan, Xiaojun Huang

**Affiliations:** ^1^ Division of Hematology/Oncology, Department of Pediatrics, The Seventh Affiliated Hospital, Sun Yat-sen University, Shenzhen, China; ^2^ Beijing Key Laboratory of Hematopoietic Stem Cell Transplantation, Peking-Tsinghua Center for Life Science, Research Unit of Key Technique for Diagnosis and Treatment of Hematologic Malignancies, National Clinical Research Center for Hematologic Disease, Peking University People’s Hospital, Peking University Institute of Hematology, Beijing, China

**Keywords:** haploidentical, hematopoietic stem cell transplantation, pediatric, acute megakaryoblastic leukemia, *de novo*

## Abstract

**Background:**

AMKL without DS is a rare but aggressive hematological malignant disease in children, and it is associated with inferior outcomes. Several researchers have regarded pediatric AMKL without DS as high-risk or at least intermediate-risk AML and proposed that upfront allogenic hematopoietic stem cell transplantation (HSCT) in first complete remission might improve long-term survival.

**Patients and method:**

We conducted a retrospective study with twenty-five pediatric (< 14 years old) AMKL patients without DS who underwent haploidentical HSCT in the Peking University Institute of Hematology, Peking University People’s Hospital from July 2016 to July 2021. The diagnostic criteria of AMKL without DS were adapted from the FAB and WHO: ≥ 20% blasts in the bone marrow, and those blasts expressed at least one or more of the platelet glycoproteins: CD41, CD61, or CD42. AMKL with DS and therapy related AML was excluded. Children without a suitable closely HLA-matched related or unrelated donor (donors with more than nine out of 10 matching HLA-A, HLA-B, HLA-C, HLA-DR, and HLA-DQ loci), were eligible to receive haploidentical HSCT. Definition was adapted from international cooperation group. All statistical tests were conducted with SPSS v.24 and R v.3.6.3.

**Results:**

The 2-year OS was 54.5 ± 10.3%, and the EFS was 50.9 ± 10.2% in pediatric AMKL without DS undergoing haplo-HSCT. Statistically significantly better EFS was observed in patients with trisomy 19 than in patients without trisomy 19 (80 ± 12.6% and 33.3 ± 12.2%, respectively, P = 0.045), and OS was better in patients with trisomy 19 but with no statistical significance (P = 0.114). MRD negative pre-HSCT patients showed a better OS and EFS than those who were positive (P < 0.001 and P = 0.003, respectively). Eleven patients relapsed post HSCT. The median time to relapse post HSCT was 2.1 months (range: 1.0–14.4 months). The 2-year cumulative incidence of relapse (CIR) was 46.1 ± 11.6%. One patient developed bronchiolitis obliterans and respiratory failure and died at d + 98 post HSCT.

**Conclusion:**

AMKL without DS is a rare but aggressive hematological malignant disease in children, and it is associated with inferior outcomes. Trisomy 19 and MRD negative pre-HSCT might contribute to a better EFS and OS. Our TRM was low, haplo-HSCT might be an option for high-risk AMKL without DS.

## 1 Introduction

Acute megakaryoblastic leukemia (AMKL) is categorized as acute myeloid leukemia (AML) M7 in the FAB classification (1) and defined by differentiation as blasts expressing at least one or more of the platelet glycoproteins: CD41 (glycoprotein IIb), CD61(glycoprotein IIIa), or CD42b (glycoprotein Ib) in the WHO classification (2). It is generally divided into three distinct groups: children with Down syndrome (DS), children without DS, and adult AMKL. Pediatric AMKL is the most common subtype of AML in the DS population, and it usually harbors somatic mutations of GATA. AMKL with DS in childhood can achieve nearly 80% 10-year overall survival while undergoing low-intensity chemotherapy (3). In contrast, pediatric AMKL without DS is a rare subtype and accounts for about 7.3%–8.7% of AML in separate large cohorts (4–7). Since pediatric AMKL without DS is thought to be often associated with myelofibrosis, complex karyotype, unfavorable fusion genes, and early disease onset (< 1 year old), dismal clinical outcomes have been observed for decades.

Several researchers have regarded pediatric AMKL without DS as high-risk or at least intermediate-risk AML and proposed that upfront allogenic hematopoietic stem cell transplantation (HSCT) in first complete remission might improve long-term survival. Several articles have reported superior or comparable event-free survival (EFS) and overall survival (OS) than chemotherapy alone (4, 5, 8, 9). Most of these studies chose a busulfan/cyclophosphamide-based conditioning regimen and matched sibling donor (MSD) or matched unrelated donor (MUD). However, MSD or MUD is quite challenging in China due to the one-child policy and limited China Marrow Donor Program resources. Over the past 10 years, HLA-haploidentical-related donor HSCT (haplo-HSCT) has been fully developed for a variety of hematological disorders (10, 11).

Haploidentical HSCT has been reported to have promising OS and EFS for acute leukemia in children with both AML and acute lymphoblastic leukemia, owing to a stronger graft-versus-leukemia effect (11–14). For patients with AMKL but not DS who lack matched donors, alternative donor transplantation should be evaluated. However, to date, there are no documented reports on haplo-HSCT for the treatment of pediatric AMKL without DS. In this article, we report the safety and efficacy of haplo-HSCT in treating 25 pediatric patients with AMKL without DS.

## 2 Method

### 2.1 Patients

Twenty-five pediatric (< 14 years old) AMKL patients without DS who underwent haploidentical HSCT in the Peking University Institute of Hematology, Peking University People’s Hospital from July 2016 to July 2021 were enrolled in this study. Parents or guardians of patients were fully informed and signed consent before HSCT. The diagnostic criteria of AMKL without DS were adapted from the FAB and WHO (2, 15): ≥ 20% blasts in the bone marrow, and those blasts expressed at least one or more of the platelet glycoproteins: CD41, CD61, or CD42. Karyotype analysis was conducted at initial diagnosis. Of the 25 patients, cytogenetic profiles were available for 22 (88%) patients. The remaining three patients were not evaluated for different reasons: two with normal karyotype but< 20 analyzed metaphases, and one for whom information was not available. A panel of recurrent fusion genes in AML was developed in our institution and was performed on 60% (15/25) patients. Next-generation sequencing (NGS) was performed for 68% (17/25) patients. This study was conducted in accordance with the Declaration of Helsinki. The study was approved by the Ethics Committee of the Peking University People’s Hospital.

### 2.2 Donor selection and grafts

Children without a suitable closely HLA-matched related or unrelated donor (donors with more than nine out of 10 matching HLA-A, HLA-B, HLA-C, HLA-DR, and HLA-DQ loci), were eligible to receive haploidentical HSCT. High-resolution typing for HLA was conducted for both the patients and close family members for assessment of haploidentical-related donors, and an appropriate donor was selected according to an algorithm in our institute (16). Of the 25 patients, 19 received bone marrow (BM) and peripheral blood stem cells (PBSCs) as the stem cell sources, whereas six patients received only PBSCs due to the quarantine regulations for COVID-19. G-CSF (5 µg/kg per day; filgrastim) was used to mobilize the bone marrow (G-BM) and peripheral blood (G-PB). Both the G-BM (collected on day 0, after 4 days of G-CSF treatment) and G-PB (collected on day 1, after 5 days of G-CSF treatment) cells were collected.

### 2.3 Conditioning and GVHD prophylaxis

A modified busulfan/cyclophosphamide plus antithymocyte globulin (ATG) was used as the conditioning regimen in HID-HSCT, consisting of cytarabine (4 g/m2 × 2d, on days −10 to −9), busulfan (4 mg/kg per day orally on days −8 to −6 before January 2008, and 3.2 mg/kg per day intravenously on days −8 to −6 after that date), cyclophosphamide (1.8 g/m2 × 2d on days −5 to −4), simustine (250 mg/m2 orally once on day −3), and ATG (2.5 mg/kg per day on days −5 to −2). And 9/25 patient received additional 5 days of Decitabine 20mg/m2/d intravenously from d-14 to d-10 after January 2020. G-CSF (5 μg/kg/day) was stated for the recipients on day + 6 post-transplantation and continued until the neutrophils count was > 0.5× 10^9^ cells/L for 3 consecutive days. Graft-versus-host disease (GVHD) prophylaxis was with cyclosporine, mycophenolate mofetil, and a short course of methotrexate as previously described. The supportive care and monitoring schedule were performed as described previously (14, 17).

### 2.4 Definition, and viral reactivation

A structurally complex karyotype was defined as the presence of ≥3 chromosomal aberrations, including at least one structural aberration. OS was based on death from any cause from the date of HSCT. The events for calculating EFS included death or disease relapse from the date of HSCT. The standard definitions of relapse were used, including BM and/or extramedullary sites. Non-relapse mortality was defined as death without relapse following HSCT. Neutrophil engraftment was defined as an absolute neutrophil count > 0.5 × 10^9/^L for 3 consecutive days. Platelet engraftment was defined as a platelet count > 20 × 10^9/^L for 7 days without transfusion. Patients had chimerism studies done with PBSCs on +1M, +2M, +3M, +4.5M, +6M, +9M, +12M and yearly thereafter. Diagnosis of GVHD was made in accordance with international criteria described in the literature (18, 19). Donor engraftment was determined by either fluorescence *in situ* hybridization for the sex chromosomes for sex-mismatched HSCT or short tandem repeats for same-sex HSCT. Viral reactivation screening was assessed by quantitative whole-blood PCR for cytomegalovirus and Epstein–Barr virus in all patients pre-HSCT and at intervals of once or twice a week post HSCT. Patients were treated with ganciclovir or foscarnet for viral reactivation if viremia was detected.

### 2.5 Statistical analysis

All results were expressed as 2-year probability or 2-year cumulative incidence (%) at the 95% confidence interval (95% CI). The OS probability and EFS were estimated by the Kaplan–Meier method. Potential prognostic factors for OS, EFS, and CIR were evaluated by log-rank test univariate analyses, and results with the P < 0.05 were considered statistically significant. The aGVHD was assessed at day 100 after HSCT, and cGVHD was assessed at the last follow-up. All statistical tests were conducted with SPSS v.24 and R v.3.6.3.

## 3 Results

### 3.1 Patients’ characteristics

Twenty-five pediatric AMKL patients (12 males and 13 females) without DS who underwent haploidentical HSCT at the Peking University People’s Hospital from July 2016 to July 2021 were included in this study. The median age of the patients at diagnosis was 25 months (range: 10–68 months), with 84% of patients being ≤ 36 months old. The median diagnosis–transplant interval was 3.6 months (range: 2–10 months). The median number of total mononuclear cells from the stem cells collected was 10.11 ×10^8/^kg (range: 7–16×10^8/^kg), and the mean number of CD34+ cells was 3.69 ×10^6/^kg (range: 1.63–26.4×10^8/^kg) in total. The median follow-up was 41.7(13.2-73.9) for survivors post HSCT.

The patients’ baseline characteristics are presented in **Table 1**. All patients met the diagnostic criteria of AMKL without DS as adapted from the FAB and WHO (2, 15). Immunophenotyping using flow cytometry (FCM) was performed at least once at initial diagnosis or for the differential diagnosis purpose. The proportion of expression of the platelet glycoproteins was described in **Table 1**. Complex karyotype was the most frequent cytogenetic abnormality, and it accounted for 64% of the patients, and details was described in **Table 1**. Although all patients achieved CR pre-HSCT, three (12%) patients still had an undesirable FCM MRD (range: 0.53%–2.84%). Central nervous system involvement was not detected in the patients included in this study.

**Table 1:** Characteristics of pediatric AMKL patients without DS undergoing haplo-transplant (n=25).

Characteristics	Patients
Gender ratio	12M/13F
Median age at diagnosis, months (range)	25 (10-68)
Median diagnosis-HSCT interval, months (range)	3.6 (2-10)
Median WBC count, ×10^9^/L (range)	9.91 (3.4-91.01)
Median Hb count, g/L (range)	88 (54-123)
Median PLT count, ×10^9^/L (range)	23 (5-219)
Immunophenotype features, no. (%)	
CD34	11 (44%)
CD41	21 (84%)
CD42	19 (76%)
CD61	32 (84%)
CD36	13 (52%)
CD56	5 (20%)
Cytogenetic features (n=22), no. (%)	
complex karyotype	16 (72.7%)
normal karyotype	3 (13.6%)
+21	7 (31.8%)
+19	10 (45.4%)
+8	5 (22.7%)
-7	1 (4.5%)
Fusion gene	
MLLr	2 (8%)
**FCM MRD negative pre-HSCT**	22 (88%)
Graft, no. (%)	
BM+PB	19 (76%)
PB	6 (24%)
Conditioning regimen, no. (%)	
Bu/Cy-ATG	16 (64%)
Dec/Bu/Cy-ATG	9 (36%)
Donor-recipient blood type match, no. (%)	14 (56%)
Median MNCs, ×10^8^/kg (range)	10.11 (7-16)
Median CD34+ cells, ×10^6^/kg (range)	3.69 (1.63-26.4)
Median duration of follow-up for survivors, months (range)	41.7 (13.2-73.9)
CMV reactivation, no. (%)	10 (40%)

NGS was performed in 68% (17/25) of the patients. Twelve of the 17 cases were without disease-related mutations. Two cases carried the KIT p.M541L mutation; there was one case with JAK2 p.V671F mutations; and one case had JAK3 p.A573V. The other three cases had the JAK2 p.Q494H, JAK2 p.R683G, or JAK2 p.V617I mutation.

### 3.2 Engraftment and GVHD

Donor and recipient sex were matched in 52% of the patients (13/25), and 76% of the patients (13/25) received G-CSF mobilized BM cells and peripheral blood stem cells, while the rest received only mobilized peripheral blood stem cells. All patients were successfully engrafted with a median time to ANC engraftment of 12 days (range: 10–16 days). Twenty-four of the 25 patients achieved platelet engraftment with a median time of 12 days (range: 10–46 days), and one patient had very early relapse and developed platelet transfusion dependency.

Of the entire cohort, two patients were diagnosed with hemorrhagic cystitis; two had autoimmune hemolytic anemia; two had human herpes virus 6 with one encephalitis episode; and one patient developed bronchiolitis obliterans. All patients survived > 10 days after HSCT and were eligible for acute graft-versus-host disease (aGVHD) evaluation. Seventeen patients (68%) experienced aGVHD. Grade I aGVHD occurred in 14 of the 17 patients, and Grade II aGVHD occurred in three. The most common involved organ was skin, and only one patient had both skin and lower gastrointestinal tract involvement. The presence of aGVHD did not affect OS (P = 0.229) and EFS (P = 0.487). One patient developed moderate cGVHD involving the gastrointestinal tract and one developed mild cGVHD involving the skin.

### 3.3 Survival outcome

**Figure 1:**
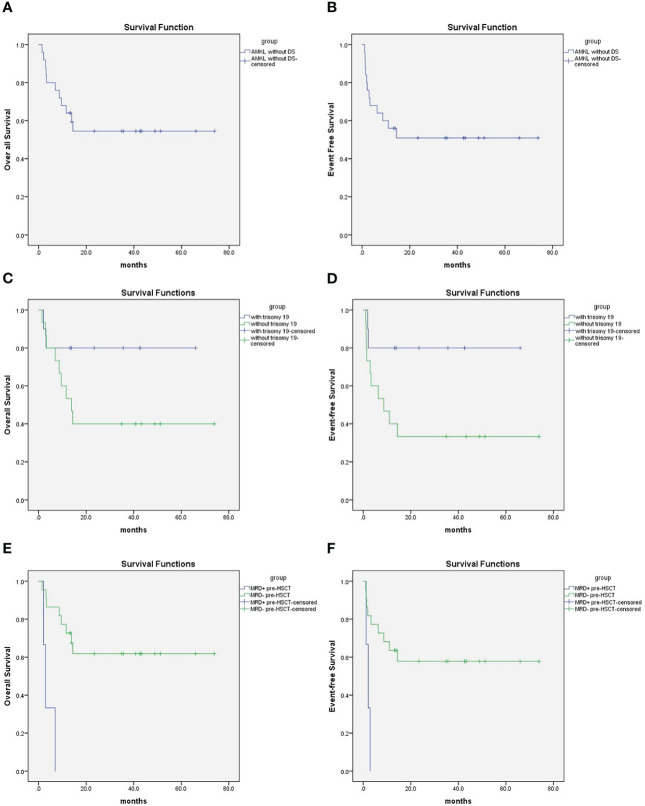
**(A)** 2-year Overall survival was 54.5 ± 10.3% for pediatric AMKL without DS undergoing haplo-HSCT; **(B)** 2-year event free survival was 50.9 ± 10.2% for pediatric AMKL without DS undergoing haplo-HSCT. **(C)** 2-year Overall survival was 80 ± 12.6% for patients with trisomy 19 and 40 ± 12.6% without trisomy 19 in pediatric AMKL without DS undergoing haplo-HSCT(*P*=0.114). **(D)** 2-year Event-free survival was 80 ± 12.6% for patients with trisomy 19 and 33.3 ± 12.2% without trisomy 19 in pediatric AMKL without DS undergoing haplo-HSCT(*P*=0.045). **(E)** 2-year Overall survival was 61.9 ± 10.7% for patients with MRD negative pre-HSCT and 0 with MRD positive pre-HSCT in pediatric AMKL without DS undergoing haplo-HSCT(*P*<0.001). **(F)** 2-year Event-free survival was57.9 ± 10.8% for patients with MRD negative pre-HSCT and 0 with MRD positive pre-HSCT in pediatric AMKL without DS undergoing haplo-HSCT (*P*=0.003).

**Table 2** describes different pretransplant characteristics and aGVHD with respect to transplant outcomes. The 2-year OS was 54.5 ± 10.3%, and the EFS was 50.9 ± 10.2% (**Figures 1A, B**). Statistically significantly better EFS was observed in patients with trisomy 19 than in patients without trisomy 19 (80 ± 12.6% and 33.3 ± 12.2%, respectively, P = 0.045) (**Figure 1D**), and OS was better in patients with trisomy 19 but with no statistical significance (P = 0.114) (**Figure 1C**). Three of the 25 patients were positive for FCM MRD pre-HSCT, and all three relapsed within 3 months post HSCT. As a result, superior EFS was achieved in the FCM MRD negative group compared with the FCM MRD positive group (57.9 ± 10.8% and 0, respectively, P = 0.003), and superior OS was achieved in the FCM MRD negative group compared with the FCM MRD positive group (61.9 ± 10.7% and 0, respectively, P < 0.001) (**Figures 1E, F**). Other factors such as age, immunophenotype, other karyotypes, graft source, and aGVHD did not reach statistical significance for both the EFS and OS.

**Table 2:** Effects of potential factors on clinical outcomes of pediatric AMKL patients without DS undergoing haplo-transplant (n=25).

	Cases	OS,%	*P* value	EFS,%	*P* value	2-year CIR	*P* value
Gender							
Male	12	48.6 ± 14.8	0.809	48.6 ± 14.8	0.924	43.1 ± 15.7	0.779
Female	13	61.5 ± 13.5		53.8 ± 13.8		46.2 ± 14.6	
Age							
<2 year old	12	57.1 ± 14.6	0.688	48.6 ± 14.8	0.825	47.4 ± 15.2	0.947
>2 year old	13	52.7 ± 14.1		53.8 ± 13.8		41.7 ± 15.1	
Immunophenotype							
CD34							
positive	11	54.5 ± 15	0.743	45.5 ± 15	0.656	45.4 ± 16.1	0.971
negative	14	52.1 ± 14.7		53.6 ± 14.5		46.2 ± 15.5	
CD36							
positive	13	61.5 ± 13.5	0.794	61.5 ± 13.5	0.647	38.5 ± 14.2	0.894
negative	12	50 ± 14.4		41.7 ± 14.2		50.0 ± 15.5	
CD41							
positive	21	50.6 ± 11.3	0.528	52.4 ± 10.9	0.389	48.7 ± 11.6	0.451
negative	4	75 ± 21.7		75 ± 21.7		25.0 ± 25.0	
CD42							
positive	19	49.7 ± 12.1	0.537	50.7 ± 12	0.795	49.3 ± 12.6	0.393
negative	6	66.7 ± 19.2		50 ± 20.4		33.3 ± 21.9	
CD61							
positive	21	59.9 ± 10.5	0.164	55.7 ± 11.2	0.069	44.3 ± 11.6	0.550
negative	4	25 ± 21.7		25 ± 21.7		50.0 ± 30.0	
CD56							
positive	5	60 ± 21.9	0.819	40 ± 21.9	0.863	40.0 ± 26.0	0.624
negative	20	52.5 ± 11.7		53.3 ± 11.6		46.7 ± 12.1	
Cytogenetic							
complex karyotypes							
Y	16	68.8 ± 11.6	0.126	68.8 ± 11.6	0.069	25.0 ± 11.2	0.033
N	9	33.3 ± 15.7		22.2 ± 13.9		77.8 ± 16.1	
Trisomy 8							
Y	15	60 ± 12.6	0.844	60 ± 12.6	0.592	16.7 ± 16.7	0.158
N	10	48 ± 16.4		37.5 ± 16.1		55.3 ± 12.9	
Trisomy 19							
Y	10	80 ± 12.6	0.114	80 ± 12.6	0.045	20 ± 13.4	0.077
N	15	40 ± 12.6		33.3 ± 12.2		60 ± 13.5	
Trisomy 21							
Y	7	42.9 ± 18.9	0.301	42.9 ± 18.9	0.343	42.7 ± 20.6	0.787
N	18	59.6 ± 11.9		54.3 ± 12.1		45.7 ± 12.6	
Normal karytypes							
Y	4	75 ± 21.7	0.314	50 ± 25	0.706	50.0 ± 30.0	0.847
N	21	51.4 ± 11.1		52.4 ± 10.9		42.9 ± 11.2	
Decitabine in conditioning regimen							
Y	9	50 ± 18.6	0.784	55.6 ± 16.6	0.83	50.0 ± 13.1	0.482
N	16	56.3 ± 12.4		50 ± 12.5		33.0 ± 16.9	
Graft source							
BM+PB	19	52.6 ± 11.5	0.792	47.4 ± 11.5	0.524	52.6 ± 11.9	0.196
PB	6	66.7 ± 19.2		66.7 ± 19.2		16.7 ± 16.7	
Donor-recipient sex match							
Y	13	52.7 ± 14.1	0.908	52.7 ± 14.1	0.776	39.6 ± 14.7	0.527
N	12	58.3 ± 14.2		50 ± 14.4		50.0 ± 15.3	
MRD status pre HSCT							
positive	3	0	<0.001	0	0.003	NA*	0.005
negative	22	61.9 ± 10.7		57.9 ± 10.8		37.6 ± 11.1	
MRD status at the end of Induction I (n=21)							
positive	12	58.3 ± 14.2	0.526	58.3 ± 14.2	0.929	NA**	NA
negative	9	66.7 ± 15.7		55.6 ± 16.6		NA	
aGVHD							
Y	17	48.8 ± 12.6	0.229	45.4 ± 12.5	0.487	48.7 ± 13.2	0.644
N	8	75 ± 15.3%		62.5 ± 17.1		37.5 ± 18.6	
CMV reactivation							
Y	10	50 ± 15.8	0.438	50 ± 15.8	0.937	40.0 ± 16.6	0.679
N	15	57 ± 13.5		51.4±·3.4		48.6 ± 14.2	

NA, Not Applicable.

NA*, All patients relapsed in this subgroup, it is not applicable in this column.

NA**, There were no TRM in MRD status at the end of Induction I (n=21), so no competing event.

### 3.4 Relapse and non-relapse related mortalities

**Figure 2:**
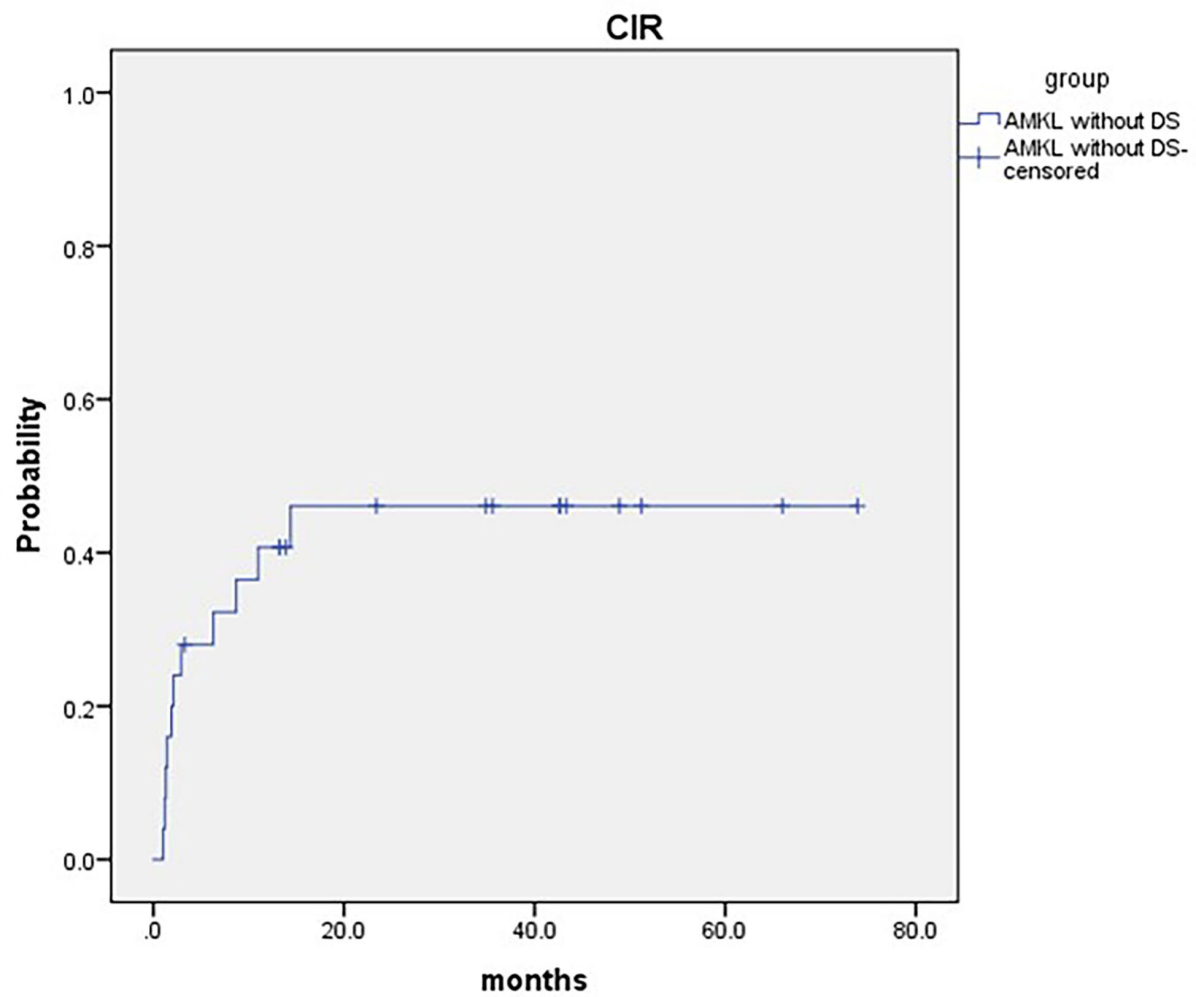
2-year cumulative incidence of relapse (CIR) was 46.1 ± 11.6% for pediatric AMKL without DS undergoing haplo-HSCT.

Eleven patients relapsed post HSCT. The median time to relapse post HSCT was 2.1 months (range: 1.0–14.4 months). The 2-year cumulative incidence of relapse (CIR) was 46.1 ± 11.6% for pediatric AMKL patients without DS undergoing haplo-HSCT (**Figure 2**). Patients with complex karyotypes had much lower CIR than those without(25.0 ± 11.2% and 77.8 ± 16.1%, respectively, P = 0.033)(**Table 2**). One patient developed bronchiolitis obliterans and respiratory failure and died at d + 98 post HSCT.

## 4 Discussion

We presented a single-center retrospective report on the clinical characteristics, outcomes, and potential prognostic factors of *de novo* pediatric AMKL patients without DS undergoing haplo-HSCT. More than two-third of our patients had a complex karyotype, which is an adverse prognostic factor in AML. Overall, our haplo-HSCT showed a comparable result with previous reports (4, 5, 20, 21). In our study, the 2-year OS was 54.5 ± 10.3% and EFS was 50.9 ± 10.2%. Inaba et al. retrospectively reviewed 490 non-DS *de novo* pediatric AMKL patients in the BFM study group and reported the 5-year EFS and OS as 43.7± 2.7% and 49.0± 2.7%, respectively, including 42% patients who received allo-HSCT (4). Thus, our results highlight that AMKL without DS is a rare but aggressive hematological malignant disease in children.

Many studies have been conducted based on the cytogenetic profile of pediatric AMKL patients without DS. What was consistent in them is that numerical abnormalities, particularly trisomy 8, trisomy 19, and trisomy 21, were more common in pediatric AMKL patients without DS than in the other AML subtypes (3, 4, 6, 7). However, when it comes to the prognostic effect on cytogenetic profiles, the conclusion seemed to be contradictory. Hara et al. reported that two recurrent chromosome abnormalities, namely CBFA2T3-GLIS2/inv(16)(p13.3q24.3) and NUP98-KDM5A/t(11;12)(p15;p13), could be identified by conventional G-banding, and CBFA2T3-GLIS2 was an independent prognostic factor for poor OS and EFS in a 44-patient Japanese pediatric cohort (7), and then de Rooij et al. validated CBFA2T3-GLIS2, NUP98/KDM5A or KMT2Ar as independent prognostic factor for poor outcome in larger cohort (22, 23). t(1;22) was first introduced in 1991 as a good prognostic factor (24) and later validated by at least two separate groups (5, 25). A recent report from Egypt demonstrated a comparable OS of 50 ± 34.6% in patients with t (1;22) and VS 52 ± 13.5% in those without t(1;22) (P = 0.43) (20). However, children’s oncology group (COG) reported that all five patients with t(1;22) survived, which might be a result of HSCT (26). The largest retrospective international pediatric AMKL study introduced a risk stratification based on cytogenetic analysis: good: abnormalities of 7p; poor: normal karyotype; −7, t(9;11), 9p: abnormalities other than (9;11); and −13, 13q−, −15, and intermediate: patients not included in the good- or poor-risk groups (4). However, the risk stratification method was not consistent with another later study (20, 27, 28). In our study, we had only one patient with t(1;22), and he survived for more than 5 years. No patient harbored −7p. Our data showed that patients with trisomy 19 had a superior EFS (80 ± 12.6% vs. 33.3 ± 12.2%, P = 0.045) and a better trend in OS and CIR, which did not reach statistical significance (80.0 ± 12.6% vs. 40 ± 12.6%, P = 0.114; 20.0% ± 13.4 vs. 60.0 ± 13.5%, P=0.077), than those without trisomy 19 (**Table 2**). Regarding complex karyotypes, trisomy 8, 21, and risk stratification by BFM (4), none of them reached statistical significance in our group (**Table 2**).

Since AMKL leukemia cell has distinct origins of megakaryocyte progenitors, the immunophenotypic profile of AMKL often showed positive for at least one of the platelet glycoproteins: CD41, CD61, or CD42, which have generally not been seen in other subtypes of pediatric AML (29, 30). It is rational to determine whether these markers could be a prognostic factor. CD36, a thrombospondin receptor, was also a surface marker for discriminate CD34− megakaryocytes and was associated with a favorable 2-year OS and EFS in several studies (6, 26, 31). However, in our study, positive status for CD34, CD36, CD41, CD42, CD56, or CD61 had no effect on prognosis.

MRD is conventionally a well-known strong prognostic factor for both OS and EFS in pediatric AML (32, 33) The Associazione Italiana di EmatoOncologia Pediatrica-AML 2002/01 trial analysis of 125 patients with *de novo* pediatric AML demonstrated a better 8-year disease-free survival (DFS) of 73.1 ± 5.6% in morphological complete remission and MRD < 0.1% groups than those in the MRD 0.1%–1% and ≥ 1% groups (P < 0.01) (34). Chen et al. reported that MRD positive at the end of induction I had a 3.81-fold hazard ratio for relapsed patients than those who were negative (35). In AMKL patients, COG reported that detection of MRD at the end of induction 2 correlated with a very poor prognosis (26), whereas Maarouf et al. did not determine a prognostic effect on the MRD level (27). In our cohort, we find that MRD negative pre-HSCT patients showed a better OS and EFS than those who were positive (P < 0.001 and P = 0.003, respectively). Notably, all three patients with MRD positive pre-HSCT relapsed and died eventually, and this conclusion might need further investigation and a larger cohort for validation.

The transplant related mortality (TRM) of our institutional haplo-HSCT protocol was low (1/25). Decitabine is a demethylation agent that has been proved to have excellent therapeutic effects in AML both pre and post HSCT (36). Most recently, several groups reported that a decitabine-intensified modified busulfan/cyclophosphamide conditioning regimen or a decitabine bridged HSCT could provide a superior OS and EFS in HSCT for AML patients (37–39). Since pediatric AMKL without DS is often associated with inferior outcomes even when undergoing HSCT, we introduced 5 days of 30 mg/m^2^/d decitabine prior to our original conditioning regimen. Unfortunately, we did not achieve a better outcome in AMKL patients—neither in all patients nor in the MRD negative subgroup (**Table 2**). A relatively short follow-up and a small sample size might be the causes of this contradictory result; therefore, further modification of the AMKL conditioning regimen may be needed for outcome improvement.

MPL mutations, which are traditionally associated with myeloproliferative neoplasms (MPNs), have been discovered in a considerable proportion of AMKL (40, 41). Our cohort and another AMKL cohort, which had a gene mutation profile in China (6), failed to identify MPL W515L and MPL T487A mutations. The JAK-STAT signaling pathway is thought to play a role in the pathogenesis of MPNs. JAK2 mutations, such as V617F, have been found recurrently in MPNs, including essential thrombocytosis. In our study, we identified five different JAK-related mutations in five individuals: JAK2 p.V671F, JAK2 p.V617I, JAK2 p.Q494H, JAK2 p.R683G, and JAK3 p.A573V. More research is needed to determine whether these MPN-related mutations have a role in the pathogenesis of pediatric AMKL without DS.

This study has some limitations. Our sample size was small, and follow-up was relatively short. However, because AMKL in childhood is a rare disease, our current study could still provide useful information to hematologists. In future, multicenter study or registry-based study may provide more convinced information.

## 5 Conclusion

AMKL without DS is a rare but aggressive hematological malignant disease in children, and it is associated with inferior outcomes. Trisomy 19 and MRD negative pre-HSCT might contribute to a better EFS and OS. Our TRM was low, haplo-HSCT might be an option for high-risk AMKL without DS.

## Data availability statement

The original contributions presented in the study are included in the article/supplementary material. Further inquiries can be directed to the corresponding authors.

## Ethics statement

The studies involving human participants were reviewed and approved by The Ethics Committee at Peking University People’s Hospital. Written informed consent to participate in this study was provided by the participants’ legal guardian/next of kin.

## Author contributions

JH and GH conceived and designed the study. JH and GH drafted the initial manuscript and analyzed the data. YC and XH reviewed the initial manuscript. YC supervised the work. JH, GH, LB, PS, YW, XZ, KL, YS, LX, JK, and CY collected and provided patient clinical data. YC and XH assigned the protocol, and critically revised the manuscript for relevant intellectual content. All authors contributed to the article and approved the submitted version.
